# Supplementary material for: Comparative multiomics analysis of cell physiological state after culture in a basket bioreactor
Source: Sci Rep. 2022 Nov 23;12:20161. doi: 10.1038/s41598-022-24687-4 (PMC9686226; doi:10.1038/s41598-022-24687-4)
Supplement: Supplementary file 1 — Supplementary Information 1. [file 41598_2022_24687_MOESM1_ESM.zip › raw data/Transcriptomic raw data/7.AS/2.dasplot/TE1vsTC1/RI/1_CHCHD6_NW_023666041.1_112261234_112261290_+@NW_023666041.1_112261234_112263469_+@NW_023666041.1_112262754_112263469_+.pdf]

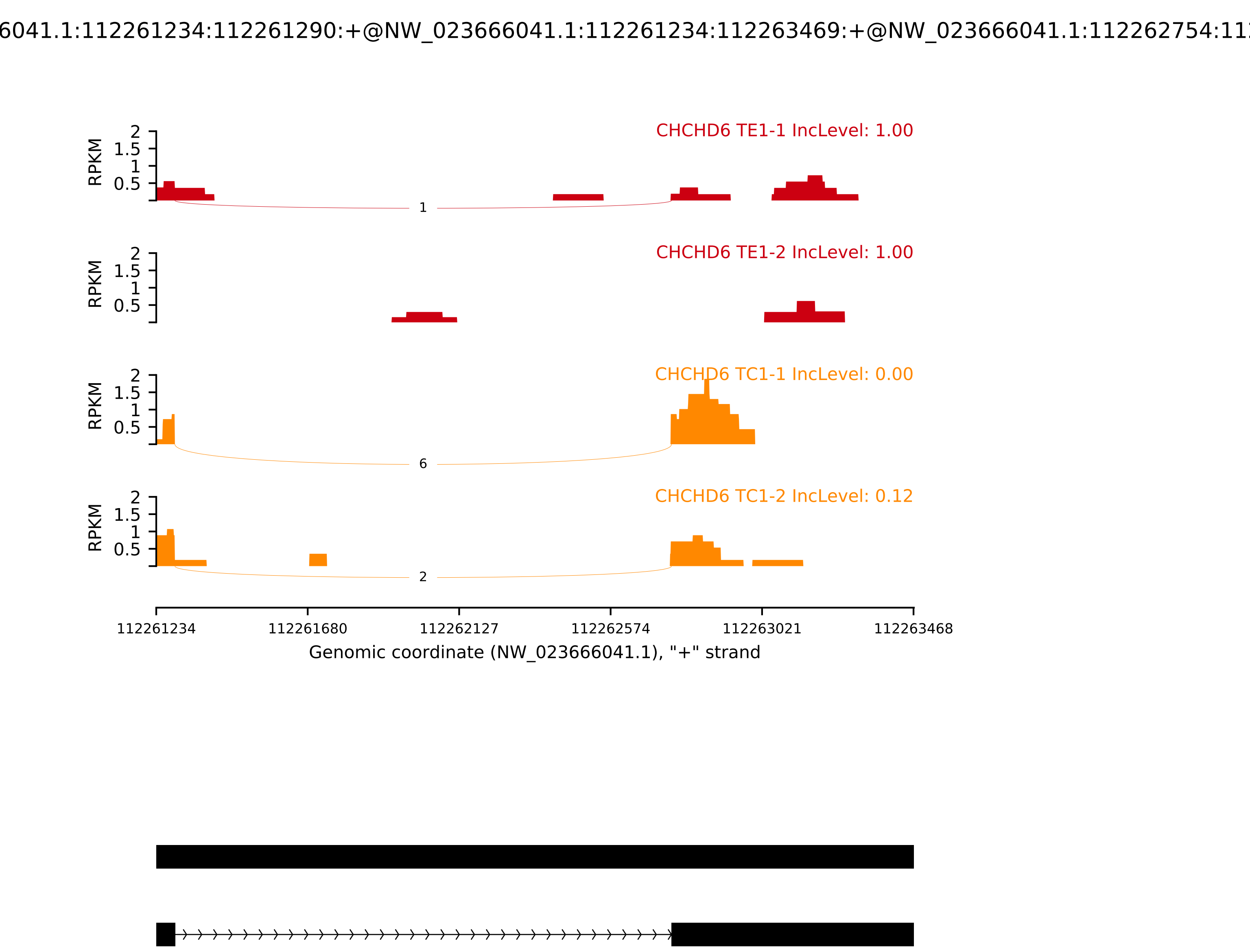
6041.1:112261234:112261290:+@NW_023666041.1:112261234:112263469:+@NW_023666041.1:112262754:112
CHCHD6 TE1-1 IncLevel: 1.00
CHCHD6 TE1-2 IncLevel: 1.00
CHCHD6 TC1-1 IncLevel: 0.00
CHCHD6 TC1-2 IncLevel: 0.12
RPKM
2
1.5
1
0.5
1
6
2
112261234
112261680
112262127
112262574
112263021
112263468
Genomic coordinate (NW_023666041.1), "+" strand
